# Determinants of willingness to pay for health insurance in later stages of the Covid-19 pandemic: findings based on the general adult population in Germany

**DOI:** 10.3389/fpubh.2025.1685694

**Published:** 2026-01-14

**Authors:** André Hajek, Hans-Helmut König

**Affiliations:** Department of Health Economics and Health Services Research, University Medical Center Hamburg-Eppendorf, Hamburg Center for Health Economics, Hamburg, Germany

**Keywords:** health insurance, willingness to pay, health satisfaction, left-wing, right-wing, income, education, coronavirus

## Abstract

**Objective:**

The aim was to examine which factors contribute to the willingness to pay (WTP) for health insurance in Germany.

**Methods:**

Cross-sectional data are taken from a large, population-based study (GESIS panel, wave 50, n = 4,447; November 2022 to January 2023). Willingness to pay for health insurance served as outcome measure. Socioeconomic, health-related, coronavirus-related, and political spectrum-related factors were included as independent variables. Multiple linear regressions with cluster-robust standard errors were used.

**Results:**

Monthly average WTP for health insurance was €258 (SD: €210). A higher WTP for health insurance was associated with being male (female vs. male: β = −0.56.6, 95% CI: −67.7 to −45.5), being older (β = 2.1, 95% CI: 1.6–2.6), higher education (e.g., intermediary school leaving certificate vs. general/subject-specific university entrance qualification: β = −67.3, 95% CI: −80.7 to −53.8), higher income group (e.g., 1,700–2,300 € vs. under 900 €: β = 79.8, 95% CI: 36.1–123.5), not being married and living together with spouse (e.g., single vs. married/partner living together: β = 28.9, 95% CI: 12.4–45.4) as well as being politically more right-wing oriented (e.g., right-wing vs. left-wing: β = 33.4, 95% CI: 4.5–62.3).

**Conclusion:**

In contrast to health- and coronavirus-related factors, socioeconomic and political spectrum-related factors were significantly associated with WTP for health insurance in Germany. Moreover, based on the average WTP, one can conclude that individuals do not fully agree with the present contributions to statutory health insurance in Germany as a whole during the Covid-19 pandemic. Future research could focus on cross-country comparisons (with varying healthcare systems and also between individualistic and collectivistic cultures).

## 1 Introduction

Similar to various other industrialized countries, demographic aging takes place in Germany. It is likely that the number of older adults will increase in Germany in the next decades. Such shifts in the demographic structure pose tremendous challenges for the German healthcare system. Maintaining support for the solidarity system of health insurance is relevant in the next decades. Therefore, knowledge about the health insurance preferences in Germany is important. Previous studies mainly focused on low-and middle-income countries without comprehensive health insurance systems (1–3).

To date, only some studies exist investigating the determinants of willingness to pay (WTP) for health insurance. Former research from Germany showed that higher WTP for health insurance is associated with lower age, being male, higher education, higher income, and higher personal healthcare costs (4–6). However, former German studies were restricted to older adults (in the federal state of Saarland - the smallest federal state that is not a city-state) or a single city (Leipzig) in Germany (4–6). Therefore, such previous results were not generalizable to the general adult population in Germany. Moreover, previous studies were conducted before the Covid-19 pandemic. More precisely, such studies used data from the late 2000s or early 2010s (4–6). It may be the case that WTP for health insurance has shifted. For example, German inhabitants may have become more aware of the importance of health in light of the pandemic [see also: (7, 8)], which may have led to an increase in the WTP for health insurance. Unlike previous research (4–6) from Germany, the self-rated political attitude and pandemic-related factors were included in the present study. Overall, our aim was as follows:

- to examine the determinants of WTP for health insurance in the German adult population against the backdrop of the Covid-19 pandemic.

Based on past research [e.g., (4, 6, 9)] we assume that a higher WTP for health insurance is positively associated with higher income in particular due to greater financial opportunities. Moreover, we assume that politically left-wing-oriented individuals have a higher WTP for health insurance compared to their counterparts. Rooted in sociological theories regarding values and solidarity, individuals with a left-wing political orientation often stress the principle of solidarity and social justice, whereby, in the end, healthy individuals pay for sick ones and individuals with a high income pay for individuals with a low income. In contrast, individuals with a right-wing political orientation often favor liberal approaches (focusing on individual responsibility, private provision and competition) (10–12). Furthermore, a rise in the awareness of health importance against the backdrop of the pandemic (7, 8) may explain why such pandemic-related factors could be positively associated with WTP for health insurance.

Overall, such knowledge may also be of relevance for potential future pandemics. Moreover, knowledge about the WTP for health insurance is crucial to ensure social cohesion during the pandemic and the current post-pandemic era.

The health care system in Germany has several noteworthy general features. Around 90% of the population is covered by social statutory health insurance (SHI), while the remaining 10% is insured through private health insurance (PHI). SHI contributions are solely income-based, irrespective of the individual's health. Although most people cannot choose PHI, it is an option for the self-employed and employees with incomes above a certain threshold (who can retain their PHI even after retirement). Unlike SHI, PHI premiums are largely determined by the individual's age and health status at the time of enrollment.

The SHI contribution per member in Germany increased over the years, which was driven by an increase of health insurance company-specific additional contributions (equally financed by employers and employees) to be able to cover costs. For example, in 2025, the health insurance contributions amount to 14.6% of income subject to general contributions plus an additional average contribution of 2.5% (depending on the health insurance company). This SHI contribution also covers spouses or children (up to 25 years old) without own income. Both PHI and SHI cover most health care expenses, including inpatient and outpatient treatments. SHI members face only small co-payments. In the PHI, deductibles vary based on the specific contract. SHI members in Germany do not receive invoices from health care providers; instead, they use a membership card, and the SHI pays the providers directly (principle of benefits in kind). Conversely, PHI members receive invoices from providers, pay them, and then get reimbursed by the PHI.

Here is also a brief overview of the pandemic situation in Germany at the time the data was collected (i.e., late 2022 to beginning of 2023): Compared to the previous pandemic years, the winter 2022/2023 can be viewed as a transitional phase toward a normalization of the situation. The far-reaching restrictions were steadily lifted.

## 2 Methods

### 2.1 Sample

Cross-sectional data (wave 50, conducted from November 2022 to January 2023) were used from the GESIS panel, a probability-based mixed-mode panel covering the general German adult population. Various topics of broad interest are included in the GESIS panel. The German-speaking population living in Germany on a permanent basis between the ages of 18 and 70 is considered as reference population. The GESIS panel is similar to the German population [with the benchmark of the German microcensus; details are presented here: (13)]. Similar to other large German surveys, the questionnaire is only available in German language. Thus, the proportion of individuals with a migration background is lower compared to the general adult German population.

In a first step, a random sample was taken from municipal population registers, and from this sample, 4,938 panelists were recruited in 2013, achieving a response rate of 86%.

Online and paper-and-pencil, were used for the initial profile survey as well as all subsequent waves. These waves took place every two months from 2013 to 2020 and every three months starting in 2021. In the GESIS panel, approximately 90% of invited participants completed each wave using the online mode, while about 85% of invited participants completed each wave using the offline mode. Additional details are provided elsewhere (13).

Data collection via the GESIS panel is possible for academic research projects. The questionnaire includes important factors such as sociodemographic information as well as tools that have been developed by the scientific community, e.g., from psychology, economics, or sociology. The evaluation of these tools is conducted through a review process, which is either internal or external. This depends on the length of the tools. More details can be found elsewhere (14). The authors of this present study suggested the WTP outcome used in this study to the GESIS panel. Following a successful internal review process by GESIS, the WTP outcome was integrated in wave 50 of the GESIS panel.

A small incentive was provided for participation (5 €). Informed consent was given by all participants included in this study. An ethics vote does not exist because it was not required (e.g., invasive methods were not used). This procedure is in accordance with local guidelines. Moreover, data collection was in accordance with the Helsinki Declaration.

### 2.2 Outcome measure: willingness to pay for health insurance

Similar to previous research (5, 6), WTP was introduced as follows: “Imagine you were not covered by health insurance and had to take out insurance on the market. In addition, the insurance policies freely available on the market would have the same scope of benefits as our statutory health insurance in Germany.” Individuals were then asked: “Taking into account your net household income: What is the maximum monthly amount you would be prepared to pay for such health insurance?” (0 €; 50 €; 100 €; 200 €; 300 €; 400 €; 500 €; 600 €; 700 €; 800 €; 900 €; 1,000 €; more than 1,000 €). Values of more than 1,000 € were converted to €1,250 to obtain definitive values for the regression analyses. Such a procedure is in accordance with previous research (4–6).

### 2.3 Independent variables

Following previous research (2, 3, 6), various independent variables were included in regression analysis. With regard to socioeconomic factors, we included age (in years), sex (men; women), marital status (single; married, living together; married, living apart; divorced; widowed), highest educational level (student; left school without certificate; graduation after a maximum of 7 years outside Germany; Secondary general school leaving certificate; intermediary school leaving certificate; Entrance qualification university of applied sciences; General/subject-specific university entrance qualification), and household net income (under 900 €; 900 up to 1,300 €; 1,300 up to 1,700 €; 1,700–2,300 €; 2,300–3,200 €; 3,200 up to 4,000 €, 4,000 up to 5,000 €; 5,000–6,000 €; 6,000 € and more). Furthermore, with regard to health-related factors, we included satisfaction with health (ranging from 1 = very unsatisfied to 7 = very satisfied; only the endpoints were labeled). With regard to coronavirus-related factors, the number of coronavirus infections (0; 1; 2; 3 or more), vaccination against coronavirus (no; at least once), and the perceived need to be hospitalized if oneself is infected with the coronavirus for the first time or again (from 1 = not at all likely to 7 = absolutely likely; solely the endpoints were labeled). With regard to political spectrum-related factors, we included the self-rated political attitude (from 0 = left to 10 = right) which is a well-established and frequently used tool. In accordance with previous research (15), we trichotomized it into: left wing (0–2), center (3–7) and right wing (8–10).

### 2.4 Statistical analysis

The characteristics of the sample were first shown. Subsequently, analogous to previous research (6) and because the outcome represents, at least approximately, a continuous variable, multiple linear regressions were performed to examine the determinants of WTP. Socioeconomic, health-related, coronavirus-related, and political spectrum-related factors were included in regression analysis. Cluster-robust standard errors were estimated (which cluster errors at the individual level).

With regard to the missing values, the great majority of independent variables had between 0 (marital status, age and sex) and 3% missing values (self-rated political attitude). The outcome had 5.4% missing values. As common (16), the income-related question had about 10% missing values. We used a full-information maximum likelihood (FIML) approach to handle missing data (17). More precisely, FIML handles missing values by including incomplete cases directly in the model estimation, computing the likelihood for each observation based solely on its observed variables. This approach uses all available data without imputation or deletion.

In a sensitivity analysis, an ordered logistic regression model was used (rather than a linear regression model). In a further analysis, sampling weights were applied. In a last sensitivity analysis, a Tobit regression was computed.

The significance level was set at 5%. StataNow 19.5 MP-Parallel Edition (Stata Corp., College Station, Texas) was used for statistical analysis.

## 3 Results

### 3.1 Sample characteristics

Characteristics of the sample are shown in Table 1. Mean age of the sample was 57.8 years (SD: 14.3 years) and 49.7% were female. Monthly average WTP for health insurance was 257.9 € (SD: 210.3 €; median: 200 €; interquartile range: 300 €). Moreover, 38.6% of the respondents had a general/subject-specific university entrance qualification. Most individuals were married/in partnership and living together (64.3%). Further details are displayed in Table 1.

**Table 1:** Sample characteristics for the analytical sample (*n* = 4,447 individuals).

**Variables**	**Mean (*SD*)/*n* (%)**
Willingness to pay for health insurance: mean (SD)	257.9 (210.3)
Sex of respondent: *N* (%)	
Male	2,236 (50.3)
Female	2,211 (49.7)
Age: mean (SD)	57.8 (14.3)
Highest level of education: *N* (%)	
Student	2 (0.0)
Left school without a certificate	29 (0.7)
Graduation after a maximum of 7 years of school attendance (abroad)	20 (0.4)
Secondary general school leaving certificate	640 (14.4)
Intermediary school leaving certificate	1,464 (32.9)
Entrance qualification university of Applied Sciences	573 (12.9)
General/subject-specific university entrance qualification	1,718 (38.6)
Household net income: *N* (%)	
Under 900 €	87 (2.2)
900–1,300 €	169 (4.2)
1,300–1,700 €	211 (5.2)
1,700–2,300 €	490 (12.1)
2,300–3,200 €	798 (19.8)
3,200–4,000 €	660 (16.4)
4,000–5,000 €	648 (16.1)
5,000–6,000 €	492 (12.2)
6,000 € and more	478 (11.9)
Marital status: *N* (%)	
Single	813 (18.3)
Married/partner living together	2,860 (64.3)
Married/partner living apart	102 (2.3)
Divorced	391 (8.8)
Widowed	281 (6.3)
Satisfaction with health (from 1 = very unsatisfied to 7 = very satisfied): Mean (*SD*)	5.0 (1.4)
Number of coronavirus infections: *N* (%)	
0	1,811 (41.2)
1	2,236 (50.9)
2	293 (6.7)
3 or more	56 (1.3)
Vaccination against coronavirus: *N* (%)	
No	313 (7.1)
At least once	4,101 (92.9)
Perceived need for hospital treatment if infected with the coronavirus for the first time or again (from 1 = not at all likely to 7 = absolutely likely): mean (SD)	2.5 (1.1)
Political spectrum: N (%)	
Left-wing	588 (14.0)
Center	3,421 (81.3)
Right-wing	201 (4.8)

### 3.2 Regression analysis

Findings of linear regressions are displayed in Table 2 (FIML was used to address missing). R^2^ was .28. Regressions showed that higher WTP was associated with being associated with being male (female vs. male, β = 56.6, *p* < .001), being older (β = 2.1, *p* < .001), higher education (e.g., intermediary school leaving certificate vs. general/subject-specific university entrance qualification, β = −67.3, *p* < .001), higher income group (e.g., 5.000–6.000 €vs. under 900 €, β = 258.7, *p* < .001), not being married and living together with spouse (e.g., widowed vs. married/partner living together, β = 56.0, *p* < .001), and being politically more right-wing oriented (e.g., right-wing vs. left-wing, β = 0.33.4, *p* < .05). In contrast, WTP was neither significantly associated with greater satisfaction with health nor with the coronavirus-related factors (i.e., number of coronavirus infections, vaccination against coronavirus, and the perceived need to be hospitalized if oneself is infected with the coronavirus for the first time or again).

**Table 2:** Determinants of willingness to pay for health insurance.

**Independent variables**	**Willingness to pay for health insurance**
Sex: female (reference category: male)	−56.55^***^ (−67.65 to −45.46)
Age	2.12^***^ (1.64 to 2.61)
Education: student (reference category: general/subject-specific university entrance qualification)	−30.07 (−242.27 to 182.14)
Left school without a certificate	−147.24^***^ (−192.51 to −101.97)
Graduation after a maximum of 7 years of school attendance (abroad)	6.30 (−128.32 to 140.91)
Secondary general school leaving certificate	−89.22^***^ (−106.80 to −71.64)
Intermediary school leaving certificate	−67.25^***^ (−80.67 to −53.84)
Entrance qualification university of Applied Sciences	−43.54^***^ (−61.77 to −25.31)
Household net income (in €)	
900–1,300 € (Reference category: Under 900 €)	10.34 (−34.29 to 54.98)
1,300–1,700 €	41.72+ (−3.35 to 86.78)
1,700–2,300 €	79.82^***^ (36.10 to 123.54)
2,300–3,200 €	118.77^***^ (74.89 to 162.65)
3,200–4,000 €	156.79^***^ (111.69 to 201.89)
4,000–5,000 €	204.55^***^ (158.54 to 250.56)
5,000–6,000 €	258.65^***^ (210.73 to 306.57)
6,000 € and more	345.48^***^ (294.87 to 396.08)
Marital status	
Single (Reference category: Married/partner living together)	28.89^***^ (12.43 to 45.35)
Married/partner living apart	59.64^**^ (23.38 to 95.89)
Divorced	32.68^***^ (13.94 to 51.43)
Widowed	56.02^***^ (36.11 to 75.94)
Satisfaction with health	1.08 (−2.91 to 5.07)
Number of coronavirus infections	
1 (Reference category: 0)	0.51 (−11.06 to 12.07)
2	−5.36 (−28.25 to 17.53)
3 or more	4.28 (−47.02 to 55.58)
Vaccination against coronavirus	
At least once (reference category: No)	19.46+ (−1.58 to 40.51)
Perceived need to be hospitalized if oneself is infected with the coronavirus for the first time or again	1.16 (−3.96 to 6.27)
Political spectrum	
Center (reference category: left-wing)	19.19^**^ (4.84 to 33.55)
Right-wing	33.39^*^ (4.48 to 62.31)
Constant	39.08 (−29.75 to 107.90)
*R* ^2^	0.28
Observations	4,447

Findings based on linear regressions.

Beta-coefficients are reported (unstandardized); 95% CI in parentheses; ^***^p < 0.001, ^**^p < 0.01, ^*^p < 0.05, + *p* < 0.10.

In a sensitivity analysis, an ordered logistic regression model was used. However, the findings remained similar (see Supplementary Table S1). In a further analysis, sampling weights were used. Findings remained virtually the same compared to the main model (see Supplementary Table S2). In a last sensitivity analysis, we calculated a Tobit regression model. Again, findings remained similar compared to our main model (see Supplementary Table S3).

## 4 Discussion

Based on data from a nationwide survey, the aim of this study was to examine the determinants of WTP for health insurance in Germany. We found a quite high average WTP for health insurance (nearly 260 € per month). Regressions showed that higher WTP for health insurance was associated with several socioeconomic factors (being male, being older, higher education and income, not being married and living with spouse) as well as being politically more right-wing oriented, whereas it was neither associated with health-related nor with coronavirus-related factors. Our findings based on nationally representative data clearly contributed to our present understanding of WTP for health insurance in Germany, mainly based on samples focusing on older adults and specific cities (4, 5, 9). Moreover, our present study adds to our current knowledge by examining the neglected factor of political attitude in the context of WTP for health insurance.

Regarding the average WTP for health insurance, previous research (6) from Germany identified a somewhat lower average WTP for health insurance (small difference—Cohen's *d* of 0.09—compared to our present findings) among the general adult population in Leipzig in the years 2011–2014. However, when comparing real values (i.e. adjusted for inflation), WTP may have fallen. We assume that these small differences could be explained by regional and period effects.

Interestingly, several sociodemographic factors were associated with higher WTP. In accordance with previous research [e.g., (4) being male was associated with higher WTP]. Perhaps, men may be aware of their less health-oriented and risk-prone lifestyle (e.g., in terms of alcohol intake, smoking behavior, use of preventive healthcare services) (18, 19). They may be pricing their lifestyle into their WTP for health insurance. Additionally, older individuals may value the great importance of health and healthcare due to their greater proximity to frailty and death. They may also have generally accumulated greater health literacy over the course of their lives, which may contribute to a higher WTP for health insurance. Moreover, individuals who are not married and live with their partner (e.g., singles) may think that they are more dependent on the healthcare system, as they cannot be cared for by their partner in the event of illness. This may lead to the fact that they value the health care system more. The higher WTP for health insurance expressed by more educated and individuals with a higher household net income may primarily be due to their larger available financial resources (not only in terms of income, but also in terms of wealth) and individuals may have a greater ability to understand the value of health insurance (4). Such findings are in accordance with previous research (2, 20).

Politically left-wing-oriented individuals reported a lower WTP for health insurance compared to their counterparts. At first glance and in light of sociological theories focusing on values and solidarity, this appears somewhat surprising given the fact that left-wing oriented individuals usually have higher preferences for redistribution, equality, and social services and tend to accept higher government taxation (10–12). These differences may be explained by differences in wealth between left-wing-oriented individuals and the other groups. Of note, it was adjusted for income in our regression analyses. However, wealth is only partly correlated with income (21) and may also be of importance in this context. Moreover, right-wing-oriented individuals may also be willing to pay more for high-quality healthcare. However, this is an exploratory explanation and thus should be examined in future research.

Unlike previous research [as an overview: (1)], health (in terms of health satisfaction) was not significantly associated with WTP in our present study. However, it may be the case that other health-related factors such as specific chronic conditions or functional impairments that severely limit activities of daily living, may be of higher relevance for WTP. Regarding the coronavirus-related factors, the number of coronavirus infections was not significantly associated with our outcome. This may be attributed to the unclear severity of the coronavirus infections. The coronavirus infections may not have shown individuals the value of appropriate healthcare. More surprisingly, the perceived need to be hospitalized if oneself is infected with the coronavirus for the first time or again was not significantly associated with WTP in our present study. It is possible that the individuals are not willing to compensate for potential future events in the sense of a higher willingness to pay now. Moreover, vaccination against coronavirus was not significantly associated (*p* < .10) with higher WTP for health insurance in our study. It may be the case that maintaining health was a key reason to vaccinate against the coronavirus for most individuals [see also: (22, 23)]. Other factors, such as pandemic fatigue or differences in the perception of risks, may also explain why the aforementioned factors were not associated with the outcome used. Factors such as altruism and empathy, which are associated with WTP (9), may be of somewhat lower relevance for the decision to vaccinate.

We acknowledge some strengths and limitations of our present work. First, it should be emphasized that a large population-based sample representative of the adult population in Germany was used. A FIML approach was used to tackle missing data. The assessment of willingness to pay (stated preferences) has a high face validity and is very similar to former studies (5, 6), which eases the comparison to previous research. However, in future studies, more complex and time-consuming strategies (5) such as bidding games could be applied. Of note, such complex models are difficult to implement in large surveys due to time restrictions. Moreover, other health-related factors such as chronic conditions, insurance status or health literacy were not assessed in this study. Such factors could be of relevance for WTP for health insurance and therefore should be included in upcoming research. Furthermore, this study has a cross-sectional design (with its inherent limitations).

In contrast to health- and coronavirus-related factors, socioeconomic and political spectrum-related factors were significantly associated with WTP for health insurance in Germany. Furthermore, based on the average WTP found in this study, one can conclude that individuals do not fully agree with the contributions to statutory health insurance in Germany as a whole (which on average amounted to more than 300 € in 2022) during later stages of the Covid-19 pandemic. From a political point of view, awareness campaigns may be beneficial, focusing, for example, on the comparatively good access and extensive range of services (often without co-payments or with only small co-payments) in the German healthcare system. This may increase the appreciation of such factors, also in monetary terms. Another approach could be to provide greater transparency about costs (e.g., medical treatment). This may raise awareness among the population of the enormous costs involved. Overall, this may ensure that the contributions are perceived as fair in the long term.

Future research could focus on cross-country comparisons (with varying healthcare systems and also between individualistic and collectivistic cultures).

## Data Availability

The datasets analyzed for this study are third-party data. Details about the data access are provided here: https://www.gesis.org/en/gesis-panel/data-access.
