# Supplementary material for: Determinants of willingness to pay for health insurance in later stages of the Covid-19 pandemic: findings based on the general adult population in Germany
Source: Front Public Health. 2026 Jan 14;13:1685694. doi: 10.3389/fpubh.2025.1685694 (PMC12847358; doi:10.3389/fpubh.2025.1685694)
Supplement: Supplementary file 1 [file Table_1.pdf]

Supplementary Table 1. Determinants of willingness to pay for health insurance. Findings based on ordered logistic regressions

Independent variables	Willingness to pay for health insurance
Sex: Female (Reference category: Male)	0.58***
	(0.52 - 0.66)
Age	1.02***
	(1.02 - 1.03)
Education: - Student (Reference category: General/subject-specific university entrance qualification)	6.00
	(0.33 - 107.82)
- Left school without a certificate	0.19***
	(0.08 - 0.46)
- Graduation after a maximum of 7 years of school attendance (abroad)	0.25*
	(0.07 - 0.82)
- Secondary general school leaving certificate	0.37***
	(0.30 - 0.45)
- Intermediary school leaving certificate	0.48***
	(0.41 - 0.55)
- Entrance qualification university of Applied Sciences	0.63***
	(0.52 - 0.75)
Household net income (in Euro): - 900 to 1300 Euro (Reference category: Under 900 Euro)	2.82***
	(1.70 - 4.66)
- 1300 to 1700 Euro	4.33***
	(2.65 - 7.07)
- 1700 to 2300 Euro	7.20***
	(4.56 - 11.35)
- 2300 to 3200 Euro	12.23***
	(7.79 - 19.21)
- 3200 to 4000 Euro	18.58***
	(11.72 - 29.48)
- 4000 to 5000 Euro	29.93***
	(18.78 - 47.70)
- 5000 to 6000 Euro	49.92***
	(30.93 - 80.57)
- 6000 Euro and more	89.95***
	(55.17 - 146.67)
Marital status: - Single (Reference category: Married/partner living together)	1.51***
	(1.26 - 1.80)
- Married/partner living apart	1.90***
	(1.30 - 2.76)
- Divorced	1.47***
	(1.19 - 1.82)
- Widowed	2.06***
	(1.61 - 2.63)
Satisfaction with health	1.00
	(0.96 - 1.05)
Number of coronavirus infections: - 1 (Reference category: 0)	1.06
	(0.94 - 1.20)
- 2	0.99
	(0.78 - 1.27)
- 3 or more	1.06
	(0.62 - 1.79)
Vaccination against coronavirus: - At least once (Reference category: No)	1.48**
	(1.16 - 1.88)
Perceived need to be hospitalized if oneself is infected with the coronavirus for the first time or again	1.00
	(0.95 - 1.06)
Political spectrum: Centre (Reference category: Left-wing)	1.18*
	(1.00 - 1.39)
- Right-wing	1.39*
	(1.03 - 1.88)
Constant	39.08
	(-29.75 - 107.90)
Pseudo R^2	.08
Observations	3,749

Odds Ratios are reported; 95% CI in parentheses; *** p<0.001, ** p<0.01, * p<0.05, + p<0.10.
