# Supplementary material for: Determinants of willingness to pay for health insurance in later stages of the Covid-19 pandemic: findings based on the general adult population in Germany
Source: Front Public Health. 2026 Jan 14;13:1685694. doi: 10.3389/fpubh.2025.1685694 (PMC12847358; doi:10.3389/fpubh.2025.1685694)
Supplement: Supplementary file 2 [file Table_2.pdf]

Supplementary Table 2. Determinants of willingness to pay for health insurance. Findings based on linear regressions (with sampling weights)

Independent variables	Willingness to pay for health insurance
Sex: Female (Reference category: Male)	-53.43*** (-78.10 - -28.76)
Age	2.06*** (0.96 - 3.15)
Education: - Student (Reference category: General/subject-specific university entrance qualification)	-1.63 (-204.95 - 201.69)
- Left school without a certificate	-140.83*** (-191.57 - -90.10)
- Graduation after a maximum of 7 years of school attendance (abroad)	35.09 (-106.71 - 176.88)
- Secondary general school leaving certificate	-83.49*** (-113.59 - -53.38)
- Intermediary school leaving certificate	-53.04*** (-82.75 - -23.33)
- Entrance qualification university of Applied Sciences	-22.67 (-88.37 - 43.02)
Household net income (in Euro): - 900 to 1300 Euro (Reference category: Under 900 Euro)	24.19 (-14.43 - 62.80)
- 1300 to 1700 Euro	58.48** (17.51 - 99.44)
- 1700 to 2300 Euro	96.05*** (57.03 - 135.06)
- 2300 to 3200 Euro	155.06** (54.74 - 255.38)
- 3200 to 4000 Euro	173.14*** (127.79 - 218.50)
- 4000 to 5000 Euro	214.99*** (167.13 - 262.86)
- 5000 to 6000 Euro	278.47*** (227.54 - 329.39)
- 6000 Euro and more	362.64*** (307.31 - 417.97)
Marital status: - Single (Reference category: Married/partner living together)	34.24 (-18.17 - 86.64)
- Married/partner living apart	58.37** (22.25 - 94.50)
- Divorced	28.70** (9.40 - 48.00)
- Widowed	59.06*** (34.96 - 83.16)
Satisfaction with health	-1.42 (-12.54 - 9.71)
Number of coronavirus infections: - 1 (Reference category: 0)	-0.25 (-13.35 - 12.86)
- 2	-8.35 (-39.16 - 22.45)
- 3 or more	11.36 (-44.21 - 66.93)
Vaccination against coronavirus: - At least once (Reference category: No)	19.29+ (-2.83 - 41.40)
Perceived need to be hospitalized if oneself is infected with the coronavirus for the first time or again	2.68 (-2.98 - 8.33)
Political spectrum: Centre (Reference category: Left-wing)	24.42* (2.39 - 46.45)
- Right-wing	35.82* (5.61 - 66.03)
Constant	18.09 (-46.03 - 82.21)
R^2	.27
Observations	4,447

Beta-coefficients are reported (unstandardized); 95% CI in parentheses; *** p<0.001, ** p<0.01, * p<0.05, + p<0.10.
