# Supplementary material for: Determinants of willingness to pay for health insurance in later stages of the Covid-19 pandemic: findings based on the general adult population in Germany
Source: Front Public Health. 2026 Jan 14;13:1685694. doi: 10.3389/fpubh.2025.1685694 (PMC12847358; doi:10.3389/fpubh.2025.1685694)
Supplement: Supplementary file 3 [file Table_3.pdf]

Supplementary Table 3. Determinants of ln willingness to pay for health insurance. Findings based on Tobit regressions.

Independent variables	Willingness to pay for health insurance
Sex: Female (Reference category: Male)	-56.27***<br>(-68.07 - -44.47)
Age	2.16***<br>(1.66 - 2.66)
Education: - Student (Reference category: General/subject-specific university entrance qualification)	115.61<br>(-235.96 - 467.17)
- Left school without a certificate	-155.93***<br>(-244.17 - -67.68)
- Graduation after a maximum of 7 years of school attendance (abroad)	-58.80<br>(-156.95 - 39.36)
- Secondary general school leaving certificate	-85.70***<br>(-105.45 - -65.95)
- Intermediary school leaving certificate	-69.82***<br>(-84.13 - -55.50)
- Entrance qualification university of Applied Sciences	-46.17***<br>(-64.64 - -27.71)
Household net income (in Euro): - 900 to 1300 Euro (Reference category: Under 900 Euro)	21.56<br>(-27.44 - 70.56)
- 1300 to 1700 Euro	52.55*<br>(5.23 - 99.86)
- 1700 to 2300 Euro	88.88***<br>(45.46 - 132.31)
- 2300 to 3200 Euro	132.27***<br>(89.61 - 174.92)
- 3200 to 4000 Euro	169.51***<br>(126.06 - 212.95)
- 4000 to 5000 Euro	216.79***<br>(172.97 - 260.60)
- 5000 to 6000 Euro	273.29***<br>(228.39 - 318.19)
- 6000 Euro and more	349.60***<br>(304.13 - 395.07)
Marital status: - Single (Reference category: Married/partner living together)	31.90***<br>(14.26 - 49.54)
- Married/partner living apart	64.46**<br>(25.74 - 103.17)
- Divorced	35.39**<br>(14.06 - 56.72)
- Widowed	51.34***<br>(26.00 - 76.68)
Satisfaction with health	0.32<br>(-3.86 - 4.51)
Number of coronavirus infections: - 1 (Reference category: 0)	2.31<br>(-10.15 - 14.76)
- 2	-5.73<br>(-30.61 - 19.15)
- 3 or more	6.57<br>(-44.26 - 57.41)
Vaccination against coronavirus: - At least once (Reference category: No)	23.20<br>(-0.62 - 47.01)
Perceived need to be hospitalized if oneself is infected with the coronavirus for the first time or again	-0.25<br>(-5.75 - 5.26)
Political spectrum: Centre (Reference category: Left-wing)	18.40*<br>(1.91 - 34.90)
- Right-wing	29.92<br>(-0.42 - 60.26)
Constant	32.84<br>(-34.46 - 100.15)
R^2	.02
Observations	3,749

Beta-coefficients are reported (unstandardized); 95% CI in parentheses; *** p<0.001, ** p<0.01, * p<0.05, + p<0.10.
